# Supplementary material for: Language interpretation and translation in emergency care: A scoping review protocol
Source: PLoS One. 2024 Nov 19;19(11):e0314049. doi: 10.1371/journal.pone.0314049 (PMC11575777; doi:10.1371/journal.pone.0314049)
Supplement: S2 Appendix — (DOCX) [file pone.0314049.s002.docx]

Appendix 2 Example search strategy

**Ovid MEDLINE(R) ALL <1946 to May 14, 2024>**

Date searched: May 15, 2024

1 Translating/ 5971

2 Translations/ 6884

3 (translator* or interpreter* or (translat* and (refugee* or migrant worker* or immigrant* or newcomer* or english proficiency or "english as a second language" or "non-native speaker*" or foreign language* or "non-english speak*")) or (translat* adj6 (speak* or language* or speech or conversation* or bilingual or multilingual or multi-lingual or google or app or apps or application* or smartphone* or "smart phone*" or "cell phone*" or "mobile phone*" or "mobile device*" or tablet or tablets or ipad* or english or spanish or hispanic* or french or german or chinese or japanese or tagalog or portugese or mandarin or cantonese or hindi or russian or ukrainian or arabic or urdu or bengali or indonesian or persian or farsi or dutch or polish or punjabi or korean or romanian or cree or hungarian or greek or italian or tamil or vietnamese or serbian or croatian or somali or gujarati or hindustani)) or (translat* adj3 (tool or tools or service or services))).mp. 35053

4 1 or 2 or 3 41623

5 Emergency Treatment/ or Emergency Medicine/ or emergency medical services/ or emergency room visits/ or exp emergency service, hospital/ or emergency services, psychiatric/ or trauma centers/ or triage/ or exp Evidence-Based Emergency Medicine/ or exp Emergency Nursing/ or Emergencies/ or (emergicent* or casualty department* or triage or ((emergenc* or ED) adj2 (room* or accident or ward or wards or unit or units or department* or physician* or doctor* or nurs* or treatment* or presentation or visit or visits or setting or patient or patients or medicine or care)) or (trauma adj1 (cent* or care))).mp. 369807

6 4 and 5 643

7 limit 6 to (comment or editorial or letter or news or newspaper article) 9

8 6 not 7 634
