# Supplementary material for: Language interpretation and translation in emergency care: A scoping review protocol
Source: PLoS One. 2024 Nov 19;19(11):e0314049. doi: 10.1371/journal.pone.0314049 (PMC11575777; doi:10.1371/journal.pone.0314049)
Supplement: S3 Appendix — (DOCX) [file pone.0314049.s003.docx]

**Data Extraction Form**

**Study characteristics**

| Study ID | Free text |
| --- | --- |
| DOI | Free text |
| First author | Free text |
| Year of publication | Free text |
| Country where study was conducted | Free text |
| Interventional or observational | Interventional, observational |
| If interventional, what is the study design | Cross-sectional study, Non-randomized controlled trial, Randomized controlled trial, Case-control study, Nested case-control study, Before-after study, Interrupted time series (without comparison group), controlled before-after study, Interrupted time series (with comparison group), Non-concurrent cohort study, Prospective cohort study, Retrospective cohort study |
| If interventional, what is the intervention(s) of interest | Free text |
| If observational, is data collection occurring prospectively or retrospectively | Prospective, retrospective, mixed, other (specify) |
| If comparison of groups, what are they | Free text |
| Data collection methods (select all) | Chart review, qualitative, survey, in-situ observation, other (specify) |

**Context characteristics**

| Number of sites | Free text |
| --- | --- |
| Setting (select all) | General ED, adult ED, pediatric ED, urgent care, pre-hospital setting, other (specify) |

**Interpretation/translation tool characteristics**

| Modality (select all) | In-person interpreter, video interpreter, phone interpreter, website, computer program, phone app, artificial intelligence, written document/questionnaire, ad hoc healthcare worker, ad hoc family/friend, not specified, other (specify) |
| --- | --- |
| Type of interpretation/translation | Simultaneous interpretation, consecutive interpretation, sight translation, dynamic translation, fixed-phrase translation, not specified, other (specify) |
| Additional description of interpretation/translation tool | Free text |
| Directionality | Unidirectional, bidirectional, not specified |
| Language options | Free text |
| Stage of clinical care (select all) | Pre-hospital, registration, triage, nurse assessment, physician assessment, reassessments, discharge, admission, not specified, other (specify) |
| Personnel utilizing tool | Nurse, physician, allied health worker, not specified, other (specify) |

**Sample characteristics**

| Study includes (select all) | Patients/family, healthcare workers, interpreters, other (specify) |
| --- | --- |
| Inclusion criteria for study | Free text |
| Exclusion criteria for study | Free text |
| Other information about sample | Free text |

Patient/Family characteristics

| Total number | Free text |
| --- | --- |
| Patient ages | Adult (>=18yo), Children (<18yo), Both |
| Gender (select all) | Male, Female, Non-Binary |
| Family members involved | Yes/No |
| If pediatric (<18yo) patients, were patients themselves asked | Yes/No/Not specified |
| Determination of English proficiency/preferred language | Free text |
| Languages spoken | Free text |

Healthcare worker characteristics:

| Total number | Free text |
| --- | --- |
| Role (select all) | Nurse, physician, resident, not specified, other (specify) |

Interpreter characteristics:

| Total number | Free text |
| --- | --- |
| Modality of Practice | In-Person, Video, Phone, Other |

**Effectiveness**

| Outcomes | Free text |
| --- | --- |
| Assessment of outcomes | Free text |

**Implementation**

| Outcomes | Free text |
| --- | --- |
| Assessment of outcomes | Free text |
| Facilitators | Free text |
| Barriers | Free text |
