# Supplementary material for: Language interpretation and translation in emergency care: A scoping review protocol
Source: PLoS One. 2024 Nov 19;19(11):e0314049. doi: 10.1371/journal.pone.0314049 (PMC11575777; doi:10.1371/journal.pone.0314049)
Supplement: Attachment — Submitted filename: Response to Reviewers ED Translation ScR Protocol (1).docx [file pone.0314049.s004.docx]

**Response to Reviewers: PLOS ONE (October 2024)**

*Title: “Language interpretation and translation in emergency care: a scoping review protocol”*

| **Reviewer** | **Section** | **Comment** | **Response** |
| --- | --- | --- | --- |
|  | General | Please ensure that your manuscript meets PLOS ONE's style requirements, including those for file naming. The PLOS ONE style templates can be found at:  <https://journals.plos.org/plosone/s/file?id=wjVg/PLOSOne_formatting_sample_main_body.pdf> and  <https://journals.plos.org/plosone/s/file?id=ba62/PLOSOne_formatting_sample_title_authors_affiliations.pdf> | Thank you. We have adjusted the formatting for the title page including adding corresponding author's initials after their email address. We have also edited the manuscript level 1 and 2 headers, as well as the supplemental file names as specified. |
|  |  | Thank you for stating the following in the Competing Interests section:  "Lisa Hartling is supported by a Canada Research Chair in Knowledge Synthesis and Translation and Elena Lopatina is supported by a Health System Impact Early Career Researcher Award, both from the Canadian Institutes of Health Research"  Please confirm that this does not alter your adherence to all PLOS ONE policies on sharing data and materials, by including the following statement: "This does not alter our adherence to PLOS ONE policies on sharing data and materials.” (as detailed online in our guide for authors http://journals.plos.org/plosone/s/competing-interests). If there are restrictions on sharing of data and/or materials, please state these. Please note that we cannot proceed with consideration of your article until this information has been declared.  Please include your updated Competing Interests statement in your cover letter; we will change the online submission form on your behalf. | We have updated the Competing Interests statement in the cover letter, as requested. |
|  |  | Please provide a complete Data Availability Statement in the submission form, ensuring you include all necessary access information or a reason for why you are unable to make your data freely accessible. If your research concerns only data provided within your submission, please write "All data are in the manuscript and/or supporting information files" as your Data Availability Statement. | Thank you, we have updated the Data Availability Statement in the submission form. As this manuscript represents a protocol, there is no current associated data to disclose. However, data associated with the study results will be made available as outlined. |
|  |  | Please include captions for your Supporting Information files at the end of your manuscript, and update any in-text citations to match accordingly. Please see our Supporting Information guidelines for more information: http://journals.plos.org/plosone/s/supporting-information. | We have updated the names of the Supporting Information files and the in-text citations to match accordingly. |
|  |  | Please review your reference list to ensure that it is complete and correct. If you have cited papers that have been retracted, please include the rationale for doing so in the manuscript text, or remove these references and replace them with relevant current references. Any changes to the reference list should be mentioned in the rebuttal letter that accompanies your revised manuscript. If you need to cite a retracted article, indicate the article’s retracted status in the References list and also include a citation and full reference for the retraction notice. | We have reviewed the reference list and believe it to be complete and correct. |
| 1 | General | The manuscript has a clear and sensible justification for why the scoping review should be proposed, analyzing an important issue of language barriers in emergency care among patients with LEP. The research questions are well-defined alongside the scope of mapping for partial and full existing literature on interpretation and translation services. However, some of the methodology and feasibility require further clarification to enhance this study. | Thank you for the thoughtful and constructive review, please see our responses below. |
|  | Introduction | Rationale and Research Questions: The rationale is sound as this would be a potential solution to address the need for improvement in professional service uptake, underpinning healthcare efficiency linked with patient health outcomes among LEP patients. The research questions address how to identify determinants for implementation, which is a topic of interest and importance in improving the quality of care. | Thank you for the kind review. |
|  | Methods | Rigor/Soundness of the Protocol: The protocol follows the Joanna Briggs Institute (JBI) methodology for scoping reviews, a legitimate and robust standard. The search strategy is comprehensive and appropriate for the study's focus based on multiple databases. Other areas where the protocol requires some elaboration to add rigor include:  Reviewer Discrepancies: The process of disagreement resolution between reviewers (or by consensus/third reviewer) is mentioned, but what constitutes ‘consensus’ needs more explicit detail. Additional transparency could be gained by detailing the rules for involving a third reviewer (e.g., ad hoc or predefined thresholds of no consensus).  Clarify how disagreements between reviewers will be settled (e.g., by a third reviewer or predefined thresholds) | Thank you for the thoughtful feedback. We aim to have a thorough and rigorous methodological approach to this study. We have expanded on how reviewer discrepancies will be handled: "*The two reviewers involved in any disagreement will meet to discuss and resolve through consensus. If consensus is not achieved through this process, then a third reviewer will be engaged for resolution.*" |
|  |  | Exploratory Features: Are any aspects of the analysis exploratory? Clearly describing exploratory analysis elements could increase transparency and prevent flexibility in interpretation.  Provide detailed information on exploratory data analysis elements and any assumptions used during data processing | Thank you for the thoughtful comment. As described in the data synthesis section, we will create descriptive summaries of the review results. The primary data processing step will be mapping barriers and facilitators to the Consolidated Framework for Implementation Research, which is already described. Apart from the outlined descriptive summaries, there will be no other exploratory aspects to the analysis. |
|  |  | Feasibility and Replicability of the Methodology: The paper does not elaborate enough on the feasibility of this methodology, particularly:  Non-English Studies: While non-English studies refer to online translation tools, it is unclear how these will be validated for medical context accuracy. The proposed method for non-English papers could raise concerns about reliability and practicality. Clarifying what tools will be used and how word-to-word accuracy will remain unchanged is necessary. Clear outcome measures are needed, or the results risk selective interpretation, reducing replicability.  Detail the translation process for non-English studies, including the tools used and how accuracy will be ensured | Thank you for pointing this out. We agree that this requires further clarity and have adjusted the manuscript to be more explicit. Unfortunately, due to financial limitations we are not able to pay for professional translation of included articles, however, we hope that the revised method reaches an adequate balance of accuracy and feasibility: "*Non-English texts will be included for consideration, however, due to financial limitations we will not be able to employ professional translation. As a result, we will use the team's internal resources alongside online language translation tools to translate non-English texts for selection and data extraction. Members of our study team are familiar with over 10 different languages. We will use both Google Translate and ChatGPT to translate non-English texts and compare the resulting translations. Machine translation tools provide a practical solution, however their accuracy may vary, especially in technical or medical contexts. Where discrepancies arise, they will be verified by a study team member familiar in the language of the text to ensure translation fidelity. Where no study team members are familiar with the language, we will ask a colleague who is familiar with the language to verify. In the event this is not possible we will report this as such in the limitations of the analysis.*" |
|  |  | Data Extraction and Outcome Measures: More details are needed on how data will be extracted and how the effectiveness of different translation modalities will be assessed. | Thank you for the suggestion. Our scoping review is focused on examining the types of outcomes used for studying interpretation/translation modalities but will not be extracting or assessing results of the included studies (e.g. impact of a particular modality on specific outcomes). We have added a line under the Data synthesis subheading to better clarify this distinction. This type of outcome/results analysis may follow as a next step in the form of a systematic review, based on what our scoping review identifies. |
|  | Data | Data Availability: The manuscript does not clearly state where the review data will be made available, which is essential for compliance with PLOS ONE’s data policy.  Clearly state where the data underlying the review will be publicly available to comply with PLOS ONE’s data policy  Availability of Data and Materials: The manuscript does not report primary data or mention where review-related data will be made publicly available. This is necessary to satisfy PLOS ONE's data availability policy. Specify if it will be included in a public repository or as additional files (if applicable). | Thank you for pointing this out. We appreciate the values underlying open data and open science. There is no primary data related to this manuscript as it is a protocol. However, we have updated the data availability statement included in the submission to note that the data included in the review will be made publicly available at the time of publication of the review results. |
|  | Conclusions | Conclusions: Although the protocol addresses a relevant issue, more details on the feasibility of the methodology, especially handling non-English studies and data availability, are necessary. Addressing these concerns is crucial to ensure the study is feasible and reproducible. | Thank you for the constructive suggestions, which we believe to have adequately addressed above. We wholeheartedly agree with the need to ensure the study is feasible and reproducible. |
| 2 | General | The paper entitled “Language interpretation and translation in emergency care: a scoping review protocol” is a review paper that intends to explore the challenges of language interpretation and translation in emergency care. The authors followed the e Joanna Briggs Institute (JBI) methodology to collect the intended data. Selection and seclusion criteria were described. The paper is well written and organized. All selected papers were reported and summarized properly according to the designated criteria. I do think this paper is publishable in its current status. | Thank you for the thoughtful and kind comments. |
| 3 | General | This research is a scoping research that can be used as a base to other research. Would have loved to read more in the discussion section. | Thank you for the thoughtful and kind comments. We have added a few sentences to the discussion to highlight preliminary quality improvement approaches to improve interpretation thus far as well as emerging modalities that will likely require further exploration and research. We plan to have a more extensive discussion in the manuscript that reports on the completed scoping review. |
